# Allergic Airway Disease Prevents Lethal Synergy of Influenza A Virus-Streptococcus pneumoniae Coinfection

**DOI:** 10.1128/mBio.01335-19

**Published:** 2019-07-02

**Authors:** Sean Roberts, Sharon L. Salmon, Donald J. Steiner, Clare M. Williams, Dennis W. Metzger, Yoichi Furuya

**Affiliations:** a Department of Immunology and Microbial Disease, Albany Medical College, Albany, New York, USA; Emory University

**Keywords:** *Streptococcus pneumoniae*, coinfection, influenza, interferon gamma

## Abstract

Fatal outcomes following influenza infection are often associated with secondary bacterial infections. Allergic airway disease (AAD) is known to influence severe complications from respiratory infections, and yet the mechanistic effect of AAD on influenza virus-Streptococcus pneumoniae coinfection has not been investigated previously. We examined the impact of AAD on host susceptibility to viral-bacterial coinfections. We report that AAD improved survival during coinfection when viral-bacterial challenge occurred 1 week after AAD. Counterintuitively, mice with AAD had significantly deceased proinflammatory responses during infection. Specifically, both CD4^+^ and CD8^+^ T cell interferon gamma (IFN-γ) responses were suppressed following AAD. Resistance to coinfection was also associated with strong transforming growth factor β1 (TGF-β1) expression and increased bacterial clearance. Treatment of AAD mice with IFN-γ or genetic deletion of TGF-β receptor II expression reversed the protective effects of AAD. Using a novel triple-challenge model system, we show for the first time that AAD can provide protection against influenza virus-S. pneumoniae coinfection through the production of TGF-β that suppresses the influenza virus-induced IFN-γ response, thereby preserving antibacterial immunity.

## Abstract (precis)

Asthma has become one of the most common chronic diseases and has been identified as a risk factor for developing influenza. However, the impact of asthma on postinfluenza secondary bacterial infection is currently not known. Here, we developed a novel triple-challenge model of allergic airway disease, primary influenza infection, and secondary Streptococcus pneumoniae infection to investigate the impact of asthma on susceptibility to viral-bacterial coinfections. We report for the first time that mice recovering from acute allergic airway disease are highly resistant to influenza-pneumococcal coinfection and that this resistance is due to inhibition of influenza virus-mediated impairment of bacterial clearance. Further characterization of allergic airway disease-associated resistance against postinfluenza secondary bacterial infection may aid in the development of prophylactic and/or therapeutic treatment against coinfection.

## INTRODUCTION

Both Streptococcus pneumoniae and influenza virus infections are leading causes of morbidity and mortality worldwide. A synergistic relationship between these two pathogens is well documented, as the majority of deaths during the 1918 influenza pandemic were attributed to secondary complications from S. pneumoniae infection (1, 2). Recent studies have begun to address why influenza patients become more susceptible to secondary bacterial infections (3–10), but we are far from having a complete understanding of these superinfections. Even less understood are how we can prevent increased susceptibility and what immune responses are required for protection.

The effect of asthma on mucosal immunity against respiratory pathogens has not been adequately addressed. During the influenza pandemic of 2009, asthma was the most common risk factor associated with morbidity among patients hospitalized with influenza (11). Interestingly, although asthma was associated with a higher hospital admission rate during influenza, hospitalized asthmatics were less likely to develop severe disease or to die than nonasthmatics (12, 13). It is possible that in the clinical setting, other factors, such as earlier hospital admission due to asthma exacerbation and the preadmission use of inhaled corticosteroids, positively influenced the disease outcome in asthmatic patients during the 2009 influenza pandemic (12). In addition, we recently reported using a mouse model of allergic airway disease (AAD) followed by influenza virus infection that AAD can be either beneficial or detrimental depending on the timing of viral and allergic challenge (14, 15). However, the susceptibility of AAD mice was assessed in the absence of secondary bacterial infection. As mentioned above, secondary bacterial infection is an important cause of mortality and morbidity during influenza pandemics.

In this work, we examined the influence of AAD on viral-bacterial coinfections, an approach that has not been documented previously. Our results demonstrate that ovalbumin (OVA)- or house dust mite (HDM)-mediated AAD confers resistance against influenza-S. pneumoniae coinfection in a mouse model. Counterintuitively, preceding AAD was associated with suppressed influenza-induced inflammation, and protection was dependent upon AAD-induced transforming growth factor β (TGF-β) production.

## RESULTS

### Allergic airway disease confers protection against postinfluenza secondary bacterial infection.

**FIG 1:**
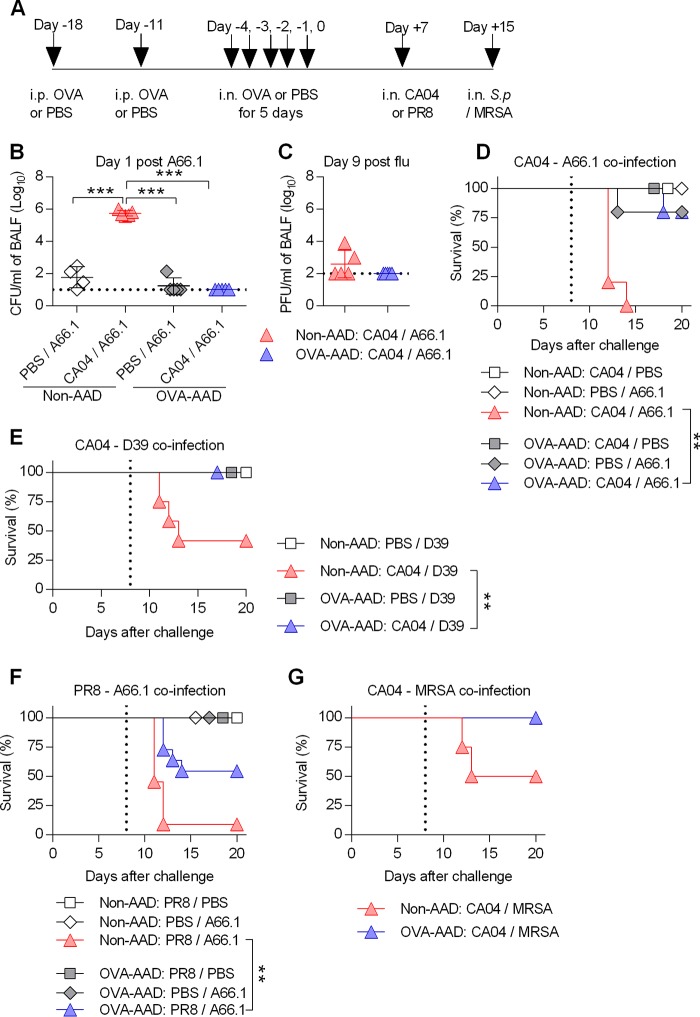
AAD mice are resistant to influenza-S. pneumoniae coinfection. (A) Schematic diagram of the OVA-induced AAD and influenza-S. pneumoniae (*S.p*) coinfection protocol. (B) BALF was harvested, and bacterial burden was assessed 1 day after S. pneumoniae A66.1 infection (*n* = 4 to 5 mice/group). The horizontal dotted line is the limit of detection. (C) The CA04 viral burden in BALF was assessed 1 day after secondary bacterial challenge by a standard plaque assay (*n* = 5 mice/group). The dotted line is the limit of detection. (D to F) OVA-AAD and non-AAD mice were singly infected or coinfected with 10 PFU of CA04 or PR8 and 2 × 10^2^ CFU of S. pneumoniae A66.1 or 1.5 × 10^4^ CFU of S. pneumoniae D39. The vertical dotted line indicates bacterial infection. (G) Non-AAD and OVA-AAD mice were coinfected with 10 PFU of CA04 and 1.7 × 10^8^ CFU of MRSA. Infected mice were monitored for survival for 20 days (*n* = 3 to 13 mice). The vertical dotted line indicates bacterial infection. **, *P* < 0.01; ***, *P* < 0.001.

To investigate the impact of AAD on viral-bacterial coinfection, we developed a triple-challenge mouse model of AAD, primary influenza infection, and secondary bacterial infection (Fig. 1A). Using this model, it was first established that influenza H1N1 A/California/4/2009 (CA04) virus-infected, non-AAD mice had defective A66.1 pneumococcal clearance at day 1 after bacterial infection (Fig. 1B), an observation consistent with our previous publications (4, 10, 16–18) and those of others (5–7, 9, 19, 20). In contrast, bacterial burden was undetectable in coinfected OVA-AAD mice and was comparable to that in control mice that were infected with S. pneumoniae alone (Fig. 1B). However, no differences in viral burden were observed in non-AAD versus OVA-AAD mice (Fig. 1C). Survival analysis showed that coinfection was lethal in non-AAD mice but not in OVA-AAD mice (Fig. 1D). This enhanced survival was not unique to the CA04 and A66.1 challenge strains, as OVA-AAD also provided protection against coinfection involving H1N1 A/Puerto Rico/8/1934 (PR8) viral or D39 bacterial strains (Fig. 1E and F). Additionally, OVA-AAD had a positive, albeit not statistically significant, effect on survival during secondary methicillin-resistant Staphylococcus aureus (MRSA) infection (Fig. 1G). The improved survival against viral-bacterial coinfection was also not dependent on the order of viral and bacterial infections, as OVA-AAD mice were also resistant to bacterial-viral coinfection (see Fig. S1 in the supplemental material). Consistent with the OVA-AAD model, HDM-AAD mice were also resistant to postinfluenza secondary bacterial challenge (Fig. S2). Importantly, the C57BL/6 mouse strain likewise exhibited improved survival during viral-bacterial coinfection following induction of OVA-AAD (Fig. S3). Thus, the positive effect of AAD was not restricted to a particular mouse strain. Based on these results, we conclude that AAD mice are resistant to influenza-bacterial coinfection and that this resistance is due to intact bacterial clearance.

10.1128/mBio.01335-19.1 FIG S1 OVA-AAD mice are resistant against bacterial-viral coinfection. (A) Diagram depicting the experimental setup. (B and C) Infected mice were monitored for weight loss (B) and survival (C) for 20 days (*n* = 4 to 5 mice/group). Dotted lines indicate viral infection. ***, *P* < 0.001. Download FIG S1, PDF file, 0.04 MB. Copyright © 2019 Roberts et al. 2019 Roberts et al. This content is distributed under the terms of the Creative Commons Attribution 4.0 International license.

10.1128/mBio.01335-19.2 FIG S2 HDM-AAD mice are resistant to viral-bacterial coinfection. (A) Schematic diagram of the HDM-induced AAD and influenza-pneumococcal coinfection protocol. (B and C) Bacterial and viral burdens in BALF harvested at day 1 after secondary bacterial challenge (*n* = 5 mice/group). The horizontal dotted line is the limit of detection. (D to F) Survival analysis of non-AAD mice and HDM-AAD mice coinfected with 10 PFU of CA04 virus, followed by either 1.5 × 10^4^ CFU of D39 (D), 2 × 10^2^ CFU of A66.1 (E), or 2 × 10^8^ CFU of MRSA (F) (*n* = 5 to 10 mice/group). Vertical dotted lines indicate bacterial infection. ***, *P* < 0.001; ****, *P* < 0.0001. Download FIG S2, PDF file, 0.05 MB. Copyright © 2019 Roberts et al. 2019 Roberts et al. This content is distributed under the terms of the Creative Commons Attribution 4.0 International license.

10.1128/mBio.01335-19.3 FIG S3 AAD confers protection in the C57BL/6 mouse strain against viral-bacterial coinfection. C57BL/6 mice were OVA treated and coinfected as described in the legend of Fig. 1A. Coinfected mice were monitored for survival for 20 days (*n* = 10 to 11 mice/group). The vertical dotted line represents secondary bacterial challenge. ****, *P* < 0.0001. Download FIG S3, PDF file, 0.02 MB. Copyright © 2019 Roberts et al. 2019 Roberts et al. This content is distributed under the terms of the Creative Commons Attribution 4.0 International license.

### AAD mice have reduced inflammatory cytokine responses during infection.

**FIG 2:**
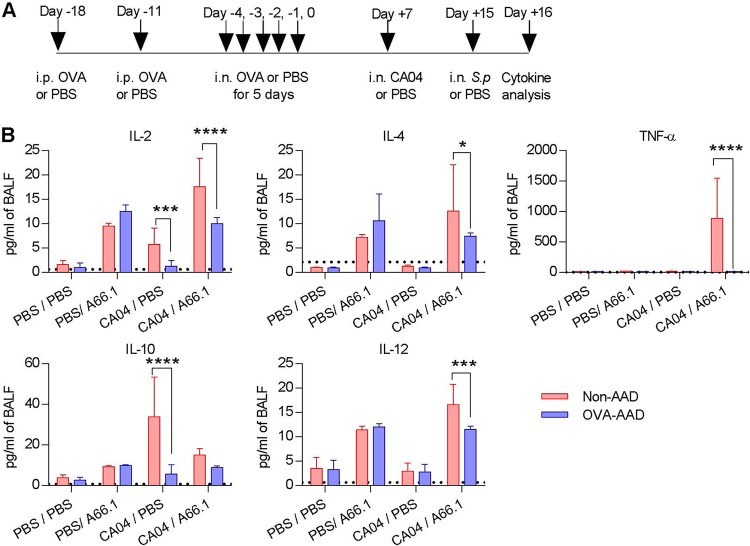
Cytokine responses are suppressed in AAD mice. (A) Experimental setup. (B) Pulmonary cytokine levels in WT BALB/c mice were quantified day 1 after secondary bacterial challenge (*n* = 4 to 10 mice/group). *, *P* < 0.05; **, *P* < 0.01; ***, *P* < 0001; ****, *P* < 0.0001.

AAD is a predominantly T helper 2 (Th2) cell-driven disease, and it is widely recognized that Th2-type cytokines play a critical role in initiating and amplifying the inflammatory response during AAD (21). Given that Th2-type cytokine production during influenza infection is linked to asthma exacerbation (22), we investigated if infection superimposed on AAD mice would have an additive or synergistic effect on inflammation (Fig. 2A and Fig. S4A). As expected, in the absence of infection, Th2- but not Th1-type cytokines were highly upregulated at day 0 after the last allergen inoculation (Fig. S4B). By day 7 after the last HDM challenge, the levels of all cytokines were below the limit of detection (Fig. S4B). In the presence of viral-bacterial coinfection, both Th1-type and Th2-type cytokines were highly upregulated in non-AAD mice (Fig. 2B and Fig. S4C). In contrast, with the exception of interleukin-5 (IL-5), these cytokine responses were significantly reduced in OVA- and HDM-AAD mice. Recent studies have demonstrated that influenza-induced inflammatory cytokines are central mediators of immune suppression after influenza infection (4–6, 8, 9, 23–25). The altered inflammatory response during influenza infection may explain why AAD mice were protected against secondary bacterial infection.

10.1128/mBio.01335-19.4 FIG S4 Th2-dominant allergic airway disease is followed by an anti-inflammatory state. (A) Diagram depicting a comorbidity model of HDM-AAD and coinfection. (B) BALF samples were collected from uninfected mice at weeks 0 and 1 after HDM challenge and assayed for various cytokine levels (*n* = 4 mice). (C) Non-AAD and HDM-AAD mice were either PBS treated or coinfected, and cytokine levels in BALF were assessed at day 1 after bacterial challenge (i.e., day +16) (*n* = 4 to 5 mice/group). *, *P* < 0.05; **, *P* < 0.01; ***, *P* < 0.001; ****, *P* < 0.001. Download FIG S4, PDF file, 0.05 MB. Copyright © 2019 Roberts et al. 2019 Roberts et al. This content is distributed under the terms of the Creative Commons Attribution 4.0 International license.

### AAD suppresses detrimental IFN-γ responses.

**FIG 3:**
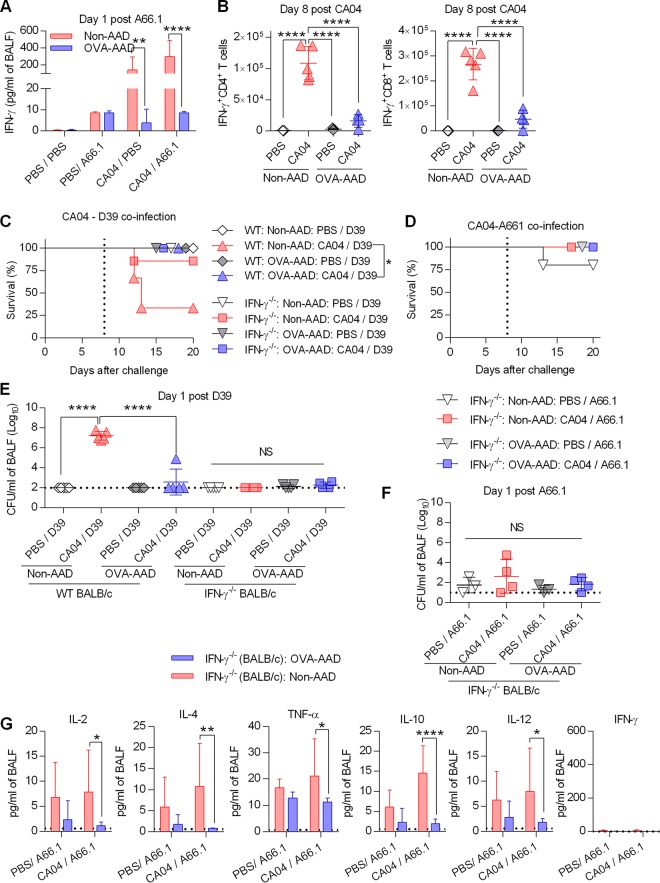
Detrimental IFN-γ is suppressed during infection in AAD mice. (A) IFN-γ levels in BALF samples harvested 1 day after secondary bacterial challenge (*n* = 4 to 10 mice/group). (B) Numbers of pulmonary IFN-γ^+^ CD4^+^ or CD8^+^ T cells at day 8 after influenza infection (*n* = 4 to 5 mice/group). (C and D) Survival analysis of WT BALB/c and IFN-γ^−/−^ mice singly infected or coinfected with CA04 and D39 (C) or A66.1 (D) (*n* = 4 to 14 mice/group). Vertical dotted lines indicate bacterial infection. (E and F) Pulmonary bacterial burdens 1 day after single infection or coinfection in WT BALB/c or IFN-γ^−/−^ mice (*n* = 3 to 5 mice/group). Horizontal dotted lines indicate the limit of detection. (G) Pulmonary cytokine levels in IFN-γ^−/−^ mice at day 1 after secondary bacterial challenge (*n* = 7 to 9 mice/group). The dotted line is the limit of detection. *, *P* < 0.05; **, *P* < 0.01; ****, *P* < 0.0001; NS, not significant.

**FIG 4:**
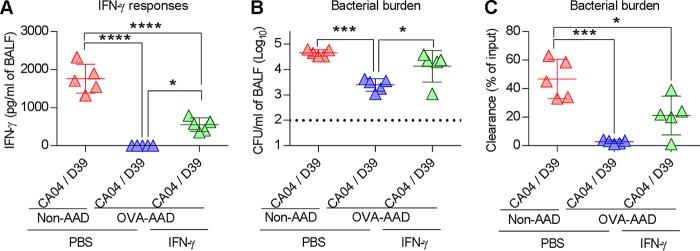
IFN-γ treatment compromises bacterial clearance in AAD mice. (A) Amount of IFN-γ in BALF 4 h after secondary bacterial challenge. Mice were i.n. treated with PBS or recombinant IFN-γ prior to a bacterial infection (*n* = 5 mice/group). (B and C) Pulmonary bacterial load, expressed as CFU per milliliter of BALF (B) or as a percentage of the input (C), 4 h after secondary bacterial challenge (*n* = 5 mice/group). The dotted line is the limit of detection *, *P* < 0.05; ***, *P* < 0.001; ****, *P* < 0.0001.

Interferon gamma (IFN-γ) has been implicated in influenza virus-induced immune suppression during secondary S. pneumoniae infection (4, 16, 26, 27). We therefore measured pulmonary IFN-γ protein concentrations. IFN-γ responses to viral single infection or viral-bacterial coinfection were significantly suppressed in AAD mice (Fig. 3A). In agreement with IFN-γ protein levels in bronchoalveolar lavage fluid (BALF), numbers of IFN-γ-positive (IFN-γ^+^) T cells were also suppressed in AAD mice (Fig. 3B and Fig. S5A). Similarly, reduced granzyme B-positive (GzmB^+^) T cell responses were observed in AAD mice (Fig. S5B). We next investigated the potential roles of IFN-γ in our triple-challenge model of AAD and coinfection. IFN-γ^−/−^ but not non-AAD wild-type (WT) mice were protected against coinfection (Fig. 3C and D and Fig. S5C), consistent with previous reports (4, 16). IFN-γ deficiency had no effect on the survival of AAD mice (Fig. 3C and D and Fig. S5). These observations suggest that while IFN-γ exerts detrimental effects on non-AAD mice during coinfection, it does not play a protective role in AAD mice. Consistent with the survival data, IFN-γ deficiency prevented inhibition of A66.1 and D39 bacterial clearance in coinfected non-AAD mice (Fig. 3E and F). The protected IFN-γ^−/−^ non-AAD mice still expressed higher levels of various cytokines than did IFN-γ^−/−^ AAD mice (Fig. 3G), suggesting that IFN-γ plays an important role in predisposing the host to secondary bacterial infections. Taken together, these data suggest that AAD mice were protected against secondary bacterial challenge due to suppression of IFN-γ responses that otherwise disabled antibacterial immunity. To directly demonstrate that suppression of IFN-γ responses was responsible for resistance against secondary bacterial challenge in AAD mice, we treated AAD mice intranasally (i.n.) with recombinant IFN-γ to increase pulmonary IFN-γ levels during coinfection (Fig. 4A). Exogenous IFN-γ significantly impaired early bacterial clearance in AAD mice (Fig. 4B and C). Therefore, the protective mechanism that exists in AAD mice may entail inhibition of detrimental IFN-γ responses.

10.1128/mBio.01335-19.5 FIG S5 A detrimental role of IFN-γ during viral-bacterial coinfection. (A) Numbers of pulmonary IFN-γ^+^ CD4^+^ or CD8^+^ T cells in HDM-AAD mice at day 8 after influenza infection (*n* = 5 mice/group). (B) Numbers of pulmonary GzmB^+^ CD4^+^ or CD8^+^ T cells at day 8 after influenza infection (*n* = 5 mice/group). (C) WT and IFN-γ^−/−^ mice were HDM treated as described in the legend of Fig. S2A and coinfected with CA04 and D39 (*n* = 4 to 5 mice/group). Mice were monitored for survival for 20 days. The vertical dotted line indicates bacterial infection. *, *P* < 0.05. Download FIG S5, PDF file, 0.04 MB. Copyright © 2019 Roberts et al. 2019 Roberts et al. This content is distributed under the terms of the Creative Commons Attribution 4.0 International license.

### Alveolar macrophage-mediated bacterial clearance is intact in AAD mice.

Since innate immune cells are involved in early bacterial clearance, we next characterized the pulmonary cellular environment to elucidate the role of innate immune cells in AAD-mediated resistance to secondary bacterial infection. Flow cytometric analysis showed that on day 7 after AAD, monocyte numbers were marginally increased in uninfected OVA-AAD mice (Fig. S6 and Fig. S7). However, upon CA04 infection, the monocyte numbers were higher in non-AAD mice than in in OVA-AAD mice. Similarly, increased numbers of neutrophils were observed in CA04-infected non-AAD mice. In contrast, levels of eosinophils were consistently higher in OVA-AAD mice on day 7 after AAD and day 8 after CA04 infection. However, eosinophil depletion using anti-IL-5 neutralizing monoclonal antibody (mAb) did not impact the survival of coinfected AAD mice (data not shown), thus eliminating the role of eosinophils in our observed protection in AAD mice. Finally, no striking differences in alveolar macrophage numbers were observed among non-AAD and OVA-AAD mice before or after CA04 infection. Based on these flow cytometry analyses, we conclude that AAD does not increase pulmonary cell numbers that may contribute to the resistance of AAD mice.

10.1128/mBio.01335-19.6 FIG S6 Gating strategy for flow cytometric identification of various innate immune cell populations. Cells were first gated based on forward scatter (FSC) and side scatter (SSC), and live cells were selected based on FVD negative staining. Live cells were defined as follows: alveolar macrophages were defined as F4/80^hi^ CD11c^hi^ Ly6G^−^ CD11b^lo^ SiglecF^+^ Ly6C^lo^ (A), eosinophils were defined as SiglecF^hi^ CD11c^lo^ F4/80^−^ CD11b^hi^ Ly6G^−^ Ly6C^−^ (B), neutrophils were defined as Ly6G^hi^ CD11b^hi^ F4/80^−^ CD11c^−^ SiglecF^−^ Ly6C^hi^ (C), and monocytes were defined as Ly6G^−^ CD11b^hi^ F4/80^lo^ CD11c^lo^ SiglecF^−^ Ly6C^hi^ (D). Download FIG S6, PDF file, 0.1 MB. Copyright © 2019 Roberts et al. 2019 Roberts et al. This content is distributed under the terms of the Creative Commons Attribution 4.0 International license.

10.1128/mBio.01335-19.7 FIG S7 AAD changes the pulmonary cellular environment. (A) Diagram depicting the timeline of OVA treatment, viral infection, and flow cytometry analysis (*n* = 8 mice/group). (B and C) Pulmonary innate immune cells were analyzed 1 week after AAD (B) and 8 days after CA04 infection (C). *, *P* < 0.05; **, *P* < 0.01; ***, *P* < 0.001. Download FIG S7, PDF file, 0.04 MB. Copyright © 2019 Roberts et al. 2019 Roberts et al. This content is distributed under the terms of the Creative Commons Attribution 4.0 International license.

**FIG 5:**
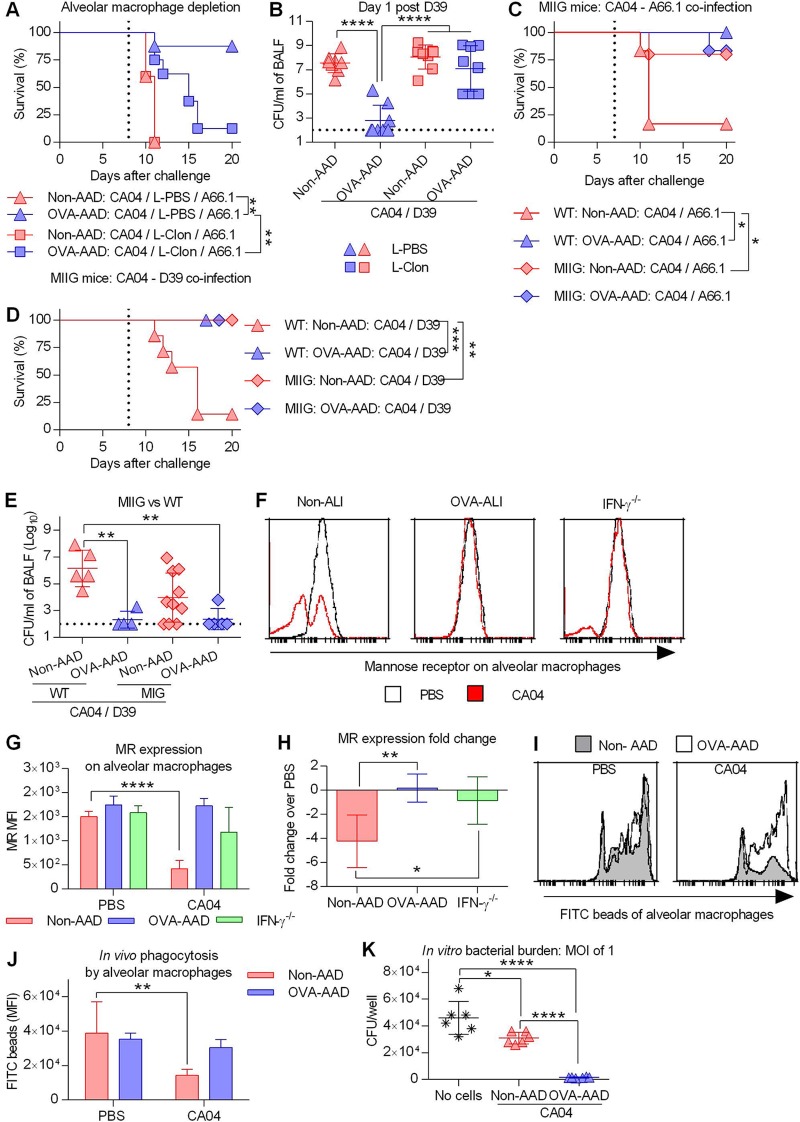
The influenza-induced defect in alveolar macrophages is absent in AAD mice. (A and B) CA04-infected mice were i.n. treated with clodronate liposomes (L-Clon) or PBS liposomes (L-PBS) on day 7 after influenza infection. CA04-infected, liposome-treated mice were coinfected with A66.1 on day 8 after influenza infection. (A) Mice were monitored for survival (*n* = 5 to 8 mice/group). The vertical dotted line indicates bacterial infection. (B) Additional mice were euthanized for assessment of bacterial burden (*n* = 8 mice/group). The limit of detection was 1 × 10^2^ CFU/ml. (C and D) WT and MIIG mice were OVA treated and coinfected as described in the legend of Fig. 1A, using A66.1 (C) or D39 (D) (*n* = 5 to 9 mice/group). Vertical dotted lines indicate bacterial infection. (E) Bacterial numbers in BALF harvested at day 1 after secondary bacterial infection (*n* = 4 to 10 mice/group). The limit of detection was 1 × 10^2^ CFU/ml. (F to H) Flow cytometric analysis of mannose receptor (MR) expression on BALF CD11c^hi^ CD11b^lo^ Ly6G^−^ cells. (F to H) Representative histograms (F), median fluorescence intensity (MFI) (G), and fold change in MFI over the PBS control (H) of MR expression at day 8 after CA04 infection (*n* = 5 to 7 mice/group). (I and J) Mock-infected or CA04-infected mice were i.n. inoculated with FITC-labeled latex beads on day 8 after influenza infection, and BALF cells were harvested for flow cytometric analysis. Representative histograms (I) and MFI (J) of the FITC signal on BALF CD11c^hi^ CD11b^lo^ Ly6G^−^ cells are shown. (K) Bacterial burden *in vitro* after 4 h of incubation with 5 × 10^4^ BALF cells at an MOI of 1 CFU of A66.1/cell. BALF cells were harvested from non-AAD and OVA-AAD mice at day 8 after influenza infection (*n* = 4 to 5 mice/group). *, *P* < 0.05; **, *P* < 0.01; ***, *P* < 0.001; ****, *P* < 0.0001.

We have previously reported that alveolar macrophages play an important role in early bacterial clearance (4, 28, 29). Indeed, depletion of alveolar macrophages during coinfection of AAD mice significantly reduced survival (Fig. 5A and Fig. S8A). Consistent with the survival data, alveolar macrophage depletion significantly increased the bacterial burden in coinfected OVA-AAD mice (Fig. 5B). It is important to note that the bacterial burden measured in clodronate-treated AAD mice was comparable to that in non-AAD mice treated with clodronate. This suggests that the observed protection in AAD mice was dependent on alveolar macrophages. The data described above demonstrated that IFN-γ played a detrimental role during coinfection and that AAD suppressed this response. We next determined if macrophages are the primary target of IFN-γ-mediated immune suppression by utilizing mice insensitive to IFN-γ (MIIG) (30). MIIG mice have a truncation in the IFN-γ receptor gene in CD68^+^ cells, rendering macrophages nonresponsive to IFN-γ. IFN-γ signaling deficiency in macrophages significantly improved the survival of coinfected non-AAD mice but had no deleterious effect on AAD mice (Fig. 5C and D and Fig. S8B). Similarly, IFN-γ signaling deficiency in macrophages resulted in fewer pulmonary bacteria in non-AAD mice although not to a statistically significant extent (Fig. 5E). We next assessed whether the difference in bacterial clearance was due to differences in macrophage-dependent phagocytosis. We measured the expression levels of mannose receptor (MR), a pattern recognition receptor that mediates nonopsonic phagocytosis by macrophages (31). CA04 infection significantly reduced mannose receptor expression in non-AAD mice (Fig. 5F to H). This reduction was absent in IFN-γ^−/−^ mice, indicating that influenza-induced IFN-γ mediates the downregulation of mannose receptor expression. OVA-AAD mice also maintained baseline expression of mannose receptor following CA04 infection, likely due to the absence of IFN-γ responses. Consistent with these phenotypic data, an *in vivo* phagocytosis assay revealed that alveolar macrophages of OVA-AAD mice maintained intact phagocytic capacity during influenza infection, while non-AAD alveolar macrophages had reduced bead uptake after CA04 infection (Fig. 5I and J). Furthermore, to evaluate the influence of influenza virus on S. pneumoniae killing activities, BALF cells were harvested on day 8 after CA04 infection and incubated with live bacteria. Bacterial burdens in culture supernatants showed that BALF cells from OVA-AAD mice can better clear S. pneumoniae *in vitro* than BALF cells from non-AAD mice (Fig. 5K). Collectively, these data suggest that influenza virus disables antibacterial functions of alveolar macrophages via IFN-γ signaling and that this detrimental immune pathway is absent in AAD mice due to suppression of IFN-γ responses.

10.1128/mBio.01335-19.8 FIG S8 IFN-γ signaling in macrophages suppresses its protective immunity. (A) Non-AAD and HDM-AAD mice were infected with CA04 virus at day 7 after HDM challenge. CA04-infected mice were treated i.n. with clodronate liposomes or PBS liposomes on day 7 after influenza infection. Mice were inoculated with D39 on day 8 after influenza infection. Mice were monitored for survival (*n* = 6 mice/group). The vertical dotted line represents secondary bacterial challenge. (B) WT and MIIG mice were HDM treated and coinfected as described in the legend of Fig. S2A. During the 20-day survival study, mice were monitored daily for mortality (*n* = 4 to 7 mice/group). The vertical dotted line represents secondary bacterial challenge. *, *P* < 0.05; **, *P* < 0.01. Download FIG S8, PDF file, 0.03 MB. Copyright © 2019 Roberts et al. 2019 Roberts et al. This content is distributed under the terms of the Creative Commons Attribution 4.0 International license.

### Correlation between upregulation of anti-inflammatory TGF-β1 and absence of detrimental IFN-γ during AAD.

**FIG 6:**
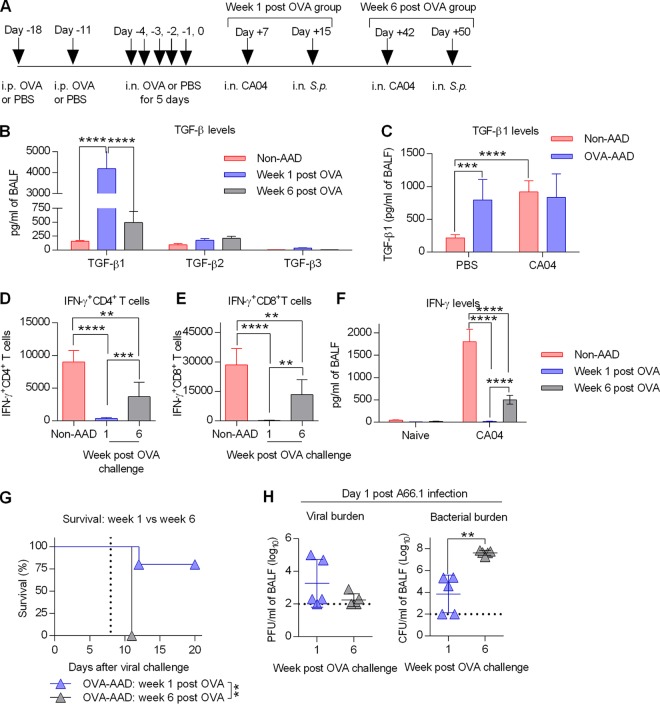
Suppressed IFN-γ responses are inversely associated with TGF-β1 responses. (A) Diagram depicting the timeline of OVA treatment and viral-bacterial coinfection. (B) TGF-β levels in BALF at week 1 or 6 after OVA challenge (*n* = 4 to 5 mice/group). (C) TGF-β1 levels in BALF in PBS-treated or CA04-infected mice at day 8 after influenza infection (*n* = 9 mice/group). (D and E) Flow cytometric analysis of IFN-γ^+^ CD4^+^ T cells (D) and IFN-γ^+^ CD8^+^ T cells (E) in BALF at day 8 after influenza infection (*n* = 5 to 8 mice/group). (F) Amount of IFN-γ in BALF at day 8 after influenza infection (*n* = 5 to 8 mice/group). (G) Survival analysis of OVA-AAD mice coinfected at week 1 or 6 after OVA challenge (*n* = 5 mice/group). The vertical dotted line indicates bacterial infection. (H) Viral and bacterial loads in OVA-AAD mice coinfected at week 1 or 6 after OVA challenge (*n* = 5 mice/group). The dotted line is the limit of detection. **, *P* < 0.01; ***, *P* < 0.001; ****, *P* < 0.0001.

We previously showed that TGF-β promotes survival of AAD mice during lethal influenza infection (14). To investigate if TGF-β also plays a role during coinfection, we determined whether there was a correlation between upregulation of TGF-β, suppression of IFN-γ, and improved survival. For this, AAD mice were examined at two time points after induction of AAD: week 1 versus week 6 after AAD (Fig. 6A and Fig. S9A). Consistent with our previous report (14), pulmonary TGF-β1 expression was highly upregulated at week 1 after AAD but returned to baseline levels at week 6 (Fig. 6B and Fig. S9B). A slight increase in TGF-β2 expression was observed in HDM-AAD mice at week 6 but not in OVA-AAD mice. TGF-β3 levels were unchanged in both OVA- and HDM-AAD mice. Following influenza infection, TGF-β1 levels were comparable between non-AAD and OVA-AAD mice (Fig. 6C). Next, AAD mice were infected with CA04 virus at week 1 or 6 for IFN-γ analysis. Robust pulmonary IFN-γ^+^ T cell responses were observed in non-AAD mice as well as in week 6 post-OVA-AAD mice but were absent in week 1 post-OVA-AAD mice (Fig. 6D and E). These flow cytometry data were confirmed by a cytokine enzyme-linked immunosorbent assay (ELISA): CA04 infection elicited strong BALF IFN-γ expression in non-AAD mice and week 6 post-OVA-AAD mice but not in week 1 post-OVA-AAD mice (Fig. 6F). As expected, AAD-mediated protection against coinfection was completely lost at week 6 (Fig. 6G and Fig. S9C). This loss of protection correlated with increased bacterial burden but not viral burden (Fig. 6H). The above-described data indicate that TGF-β1 is responsible for the suppressed IFN-γ cytokine milieu in the lungs of AAD mice, which in turn decreases susceptibility to secondary bacterial infections after influenza infection.

10.1128/mBio.01335-19.9 FIG S9 TGF-β signaling mediates resistance of HDM-AAD mice against viral-bacterial coinfection. (A) Application scheme. Mice were treated i.n. with either PBS or HDM and influenza infected at week 1 or 6 after the last HDM treatment. At day 8 after influenza infection, mice were infected with S. pneumoniae. (B) TGF-β levels were measured in BALF of uninfected mice at the indicated time points (*n* = 4 to 5 mice/group). (C) HDM-AAD mice were coinfected at week 1 or 6 after the last HDM treatment and monitored for survival (*n* = 6 to 9 mice/group). The vertical dotted line represents secondary bacterial challenge. (D) *T*β*RIIf/f-Cre* and WT control mice were HDM treated and coinfected for survival analysis (*n* = 8 to 10 mice/group). The vertical dotted line represents secondary bacterial challenge. **, *P* < 0.01; ***, *P* < 0.001; ****, *P* < 0.0001. Download FIG S9, PDF file, 0.05 MB. Copyright © 2019 Roberts et al. 2019 Roberts et al. This content is distributed under the terms of the Creative Commons Attribution 4.0 International license.

### TGF-βRII signaling mediates suppression of IFN-γ and protection against secondary bacterial challenge.

**FIG 7:**
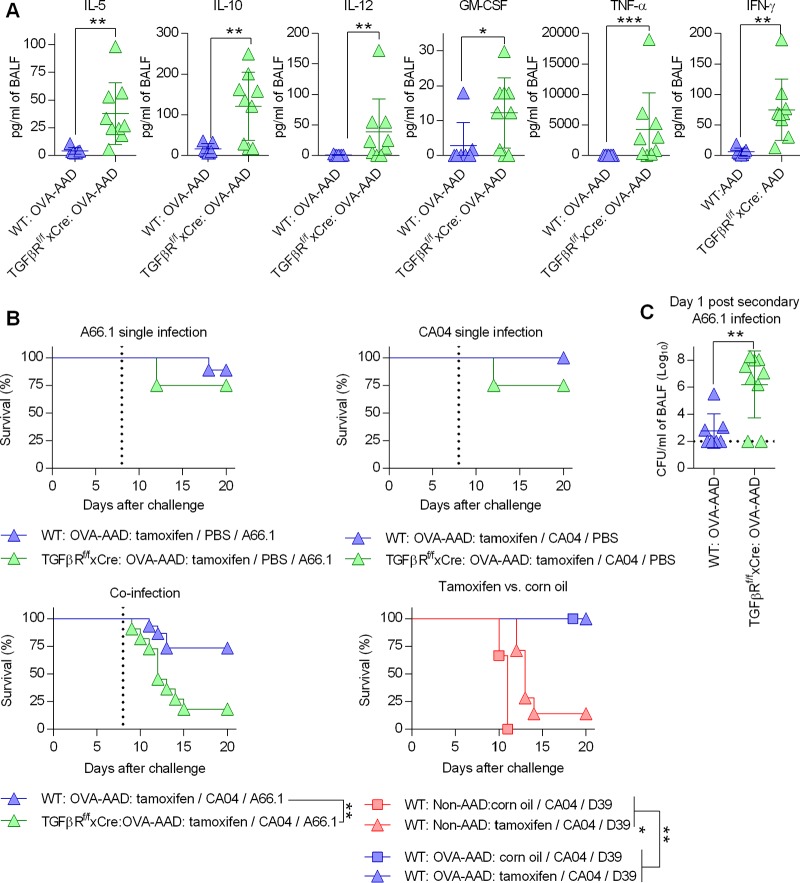
TGF-β mediates resistance of AAD mice against secondary bacterial challenge. *T*β*RIIf/f-Cre* mice and wild-type littermates were OVA treated and coinfected as described in the legend of Fig. 1A. (A) Cytokine analysis at day 1 after secondary bacterial challenge (*n* = 7 to 9 mice/group). GM-CSF, granulocyte-macrophage colony-stimulating factor. (B) Survival analysis of mice with viral infection, bacterial infection, or coinfection (4 to 10 mice/group). The vertical dotted line represents PBS or A66.1 challenge. (C) Bacterial burden at day 1 after secondary bacterial challenge (*n* = 7 to 9 mice/group). The dotted line is the limit of detection. *, *P* < 0.05; **, *P* < 0.01; ***, *P* < 0.001.

To determine whether there was a causal relationship between the above-described observations, conditional TGF-β receptor II (TGF-βRII)-deficient mice were used. Loss of TGF-βRII during coinfection significantly increased the expression of various cytokines, including IFN-γ, in OVA-AAD mice (Fig. 7A). While TGF-βRII deficiency had a minimal impact on survival during single infection, a significant reduction in survival of coinfected AAD mice was observed (Fig. 7B and Fig. S9D). TGF-βRII deficiency also caused a significant increase in the bacterial burden during coinfection in OVA-AAD mice (Fig. 7C), which likely accounts for the observed mortality.

## DISCUSSION

This is the first study to examine the impact of AAD on viral-bacterial coinfection, using a novel triple-challenge mouse model of AAD, primary influenza infection, and secondary S. pneumoniae infection. Here, we provide evidence for a novel finding in AAD mice whereby the host becomes resistant to postinfluenza bacterial infection. We conclude that AAD can transiently prevent viral-bacterial lethal synergy and thereby provide a survival advantage during secondary bacterial infection.

Using mice with a conditional deletion of TGF-βRII, we identified TGF-β as a central mediator of the observed protection. The data strongly suggest, however, that TGF-β is not an effector cytokine that directly or indirectly facilitates bacterial clearance. Rather, TGF-β reverses or prevents influenza virus-induced inhibition of pulmonary bacterial clearance. In support of this, i.n. treatment with recombinant mouse IFN-γ renders AAD mice unable to effectively control bacterial replication despite having high levels of TGF-β. Additionally, AAD-associated protection was lost following depletion of alveolar macrophages, a primary effector cell type responsible for early bacterial clearance. It is important to note that the bacterial burden measured in macrophage-depleted AAD mice was comparable to that in macrophage-depleted or -undepleted non-AAD mice. This suggests that the observed protection in AAD mice is dependent on intact alveolar macrophages and that AAD does not enhance other antibacterial pathways that could compensate for the loss of macrophages. In support of this, numbers of other phagocytic cell types, such as monocytes and neutrophils, were found to be reduced in AAD mice.

It has been shown that proinflammatory cytokine responses directed against influenza viruses are a key driver of secondary bacterial infections. In particular, IFN-γ appears to play a central role in suppressing antibacterial immunity during influenza infection (23). This detrimental IFN-γ response was found to be significantly suppressed during infection of AAD mice. However, deletion of TGF-βRII signaling unleashed IFN-γ responses in AAD mice. This was associated with the outgrowth of S. pneumoniae and the loss of a survival advantage in AAD mice. Based on these observations, it was concluded that AAD-induced TGF-β promotes survival during coinfection by suppressing detrimental IFN-γ responses. Furthermore, we investigated the downstream deleterious effects of IFN-γ signaling. The use of MIIG mice provided evidence that influenza-induced IFN-γ directly interacts with phagocytic cells to suppress antibacterial immunity. Thus, we have identified the protective immune pathway triggered by AAD: transiently heightened levels of TGF-β result in suppression of IFN-γ expression, and this in turn prevents IFN-γ–alveolar macrophage interactions that would otherwise inhibit antibacterial immunity and cause enhanced susceptibility to secondary bacterial infections.

TGF-β is known to be directly activated by microbial enzymes such as neuraminidase (NA) of influenza A virus (32–35) and of S. pneumoniae (36). One group recently reported that influenza A virus NA enhances *in vitro* bacterial adherence to cultured A549 human lung carcinoma cells, in a TGF-β signaling-dependent manner (32). Therefore, it was hypothesized that upregulation of TGF-β during influenza infection promotes secondary bacterial infection *in vivo*. Indeed, the same group reported that primary influenza infection enhances group A *Streptococcus* bacterial burden in the lungs of WT mice but not in the lungs of mice deficient in TGF-β signaling (32). Whether influenza virus NA-activated TGF-β could play a similar role in influenza-S. pneumoniae coinfection is unknown. Of note, in our model, TGF-β activation was triggered by AAD and preceded influenza infection. Thus, activation of TGF-β prior to influenza infection may be necessary to exert its protective effect during viral-bacterial coinfection. Our findings also suggest that the benefit of suppressing IFN-γ by AAD-induced TGF-β outweighs any potential detrimental effect of TGF-β-mediated bacterial adherence for coinfections involving S. pneumoniae.

Given that AAD-induced TGF-β1 responses were transient, it was not surprising that AAD-mediated protection was also transient. By week 6 after AAD, TGF-β1 levels returned to baseline, and protection against coinfection was completely lost. Similar to our mouse model, TGF-β upregulation is inducible and transient in human asthmatics (37). One longitudinal clinical study demonstrated that the concentration of TGF-β1 in BALF returns to baseline within 1 week after allergen exposure (38). Thus, it can be predicted that asthmatic episodes must precede viral infection for TGF-β to be upregulated and provide survival benefits against secondary bacterial infection. In support of this, Avila et al. (39) demonstrated that allergic subjects with experimental rhinovirus infection exhibit a significantly delayed onset of cold symptoms and a reduced duration of illness if high-dose allergen exposure preceded viral inoculation.

Consistent with our previous publication (14), this study also showed that pulmonary viral load was not exacerbated by AAD. Other investigators that have relied on a comorbidity mouse model of asthma and influenza have also reported that a preceding acute allergen challenge does not impair viral clearance (40–44). In fact, it was shown that AAD reduces pulmonary influenza viral burden (40–44), although the proposed immunological mechanisms are inconsistent among various studies. In support of these findings, a recent human study using an *ex vivo* influenza infection model of bronchial tissue explants demonstrated that viral load was reduced in bronchial biopsy specimens derived from asthmatic subjects (45). Thus, our finding that viral clearance was not impeded by prior AAD is consistent with the literature. Of note, cytolytic T cell responses were detected in our mouse model of AAD following influenza infection. It is likely that detectable, albeit reduced, levels of antiviral GzmB^+^ T cells were sufficient to clear the viral infection given that a low dose of influenza virus (10 PFU) was used in our coinfection model.

Suppression of not only IFN-γ but also the cytolytic protein GzmB in AAD mice suggests that a preceding AAD leads to a general inhibition of T cell effector functions. Our results indicate that TGF-β is playing a role in suppressing effector functions of T cells in AAD mice. This hypothesis is based on observations that deletion of TGF-βRII signaling significantly increased IFN-γ levels in the lungs of coinfected AAD mice at day 9 after viral infection, a time point that corresponds to the peak of the T cell response in influenza-infected mice. Furthermore, intracellular staining revealed that the major sources of IFN-γ during coinfection are CD4^+^ and CD8^+^ T cells in susceptible non-AAD mice. The suppressive effects of TGF-β on T cells are well established in cancer immunology (46–49). Substantial evidence exists that TGF-β can directly regulate activation, proliferation, differentiation, and survival of T cells. TGF-β signaling in T cells is mediated by TGF-βRI and -II, and the eventual activation of downstream transcription factors, such as Smad, regulates the T cell phenotype (50). This pleiotropic cytokine can also promote CD4^+^ regulatory T cell (Treg) responses. Whether the suppression of IFN-γ^+^ T cell responses in AAD mice is a direct effect of TGF-β on effector T cells or of Treg induction remains to be elucidated.

Like CA04 viral infection, preexisting AAD also conferred protection in PR8 virus-infected mice against secondary S. pneumoniae infection. This observation suggests that our result was not due to a unique phenotype of the CA04 virus but rather reflected a general phenomenon associated with influenza A virus infection in AAD mice. Furthermore, CA04-infected AAD mice were also resistant to secondary methicillin-resistant Staphylococcus aureus infection. MRSA is an emerging bacterial pathogen associated with recent seasonal and pandemic influenza. Thus, our findings may have broad application to other secondary bacterial pathogens. A better understanding of the protective immune mechanisms that exist in AAD mice is a significant first step that could eventually lead to the development of immunomodulation strategies to ameliorate detrimental immune responses.

The long-standing dogma that asthma is a risk factor for severe influenza has been challenged by recent clinical studies. Veerapandian et al. (51) conducted a systematic literature review of clinical reports on asthmatic patients during the 2009 pandemic of H1N1 virus and confirmed that asthma was a risk factor for hospitalization. However, the same authors also concluded, based on an overwhelming amount of clinical data (12, 13, 52–59), that asthmatics were less likely to develop severe influenza, as defined as intensive care unit (ICU) admittance and/or death (51). Given that 29 to 55% of deaths during the 2009 H1N1 pandemic were due to complications from secondary bacterial infections (60–62), it is possible that less severe influenza outcomes among asthmatics are due to prevention of secondary bacterial infections. Nonetheless, no data are available to support or refute this prediction, since the majority of clinical studies into the role of asthma in infection severity have focused on single pathogens. Only a limited number of studies have investigated the potential interaction between asthma and viral-bacterial coinfection. For example, Kloepfer et al. (63) recently examined coinfection in school-age children with and without asthma and concluded that asthma is not a risk factor for rhinovirus-S. pneumoniae coinfection. Unfortunately, patient samples that were positive for other viruses were excluded from their analysis. Thus, whether asthma is a risk or protective factor for influenza-S. pneumoniae coinfection is currently unknown.

A lower threshold of hospitalization for asthmatics has been proposed to explain why asthma was associated with less severe outcomes among hospitalized patients. However, Myles et al. (12) concluded that there was not a lower threshold for hospital admission for asthmatic patients since asthmatic and nonasthmatic patients presented with pneumonia at the time of admission in equal proportions. It was also noted in that clinical report that asthmatics were in fact more likely to exhibit features of severe respiratory compromise at the time of hospital admission. Thus, the improved clinical outcomes of asthmatic patients are unlikely to be the result of milder illness at the time of hospital admission. The same authors further concluded that preadmission steroid use contributed to the association of asthma with less severe clinical outcomes; however, preadmission steroid use was found to be beneficial only in asthmatic patients but not in nonasthmatic patients. This suggests that the preadmission steroid is not inherently protective against influenza. In addition, the benefit of in-hospitalization systemic steroid therapy is controversial, as some recent studies reported that steroid administration results in higher incidences of hospital-acquired bacterial pneumonia and of mortality (64–67). Thus, it is plausible that the immunosuppressive effects of preadmission steroid use may have predisposed asthmatics to influenza infections, which would explain why asthma was found to be a risk factor for increased hospitalization due to influenza. We propose that future studies should investigate the role of corticosteroids, in the context of asthma, in influencing susceptibility to coinfections. Such studies could provide a definitive answer for the controversial role of corticosteroids in influenza-infected asthmatic patients.

While epidemiological data derived from the H1N1 pandemic of 2009 support our current findings, caution is needed in extrapolating data from mice to human disease. Since mice do not naturally develop asthma, the applicability of mouse models of asthma has long been debated. Of particular concern are the lack of irreversible airway remodeling and the lack of chronicity in the acute asthma model (68, 69). As such, short-term models can be used to investigate the impact of severe acute allergic inflammation on subsequent respiratory infection but are inadequate for investigation of the relationship between chronic inflammation and host susceptibility to pulmonary pathogens. In an attempt to overcome some of the limitations of acute mouse models of asthma, a number of investigators have developed mouse models of chronic asthma by extending the period of allergen challenge (70–73). These chronic mouse models better mimic various features of human airway remodeling and therefore make it possible to study host susceptibility during the chronic phase of asthma in mice. The impact of chronic allergic inflammation on influenza-induced susceptibility to secondary bacterial infection is under investigation.

While informative epidemiological data are greatly lacking, it has been reported by various investigators that asthmatic patients exhibit defective type I IFN (IFN-I) and IFN-II responses during viral infections, as characterized *in vivo* and *ex vivo* (74–77). Coincidently, numerous mouse studies of viral-bacterial coinfection have identified IFN-I and -II as mediators of heightened sensitivity to secondary bacterial challenges (4, 6, 8, 9, 16). Thus, if these cytokines are indeed responsible for predisposing the host to secondary bacterial infections in humans, it can be extrapolated that decreased levels of IFN-I and -II in asthmatic patients would confer some level of protection during coinfection. Further research will be needed to confirm our hypothesis on the role of asthma during viral-bacterial coinfection.

The synergistic mechanisms of viral-bacterial coinfections have been investigated by a number of researchers, with the ultimate goal of developing therapeutic approaches to prevent mortality and morbidity. Most, if not all, investigators have relied on a mouse model of primary influenza infection and secondary bacterial infections (4, 6–9, 19, 20, 78–80), with the principal aims of identifying detrimental immune responses in coinfected mice and of understanding how influenza virus predisposes mice to secondary bacterial infections. The rationale behind this approach is that understanding the nature of the disadvantageous immune response may enable reversal of the immunocompromised state. In contrast, the present study focused on understanding protective elements of the immune response by examining mice that are resistant to secondary bacterial infections. Our data showing a remarkable resistance of AAD mice to coinfection were surprising and now offer a unique opportunity to understand a beneficial immune pathway that may render the host transiently resistant to secondary bacterial infections. To the best of our knowledge, a triple-challenge mouse model of asthma, primary influenza infection, and secondary pneumococcal infection has not been previously documented in the literature. Further characterization of AAD-associated resistance against viral-bacterial coinfection may aid in the development of prophylactic and/or therapeutic treatment against coinfection.

## MATERIALS AND METHODS

### Mice.

Adult 6- to 8-week-old BALB/c and C57BL/6 mice were purchased from Charles River Laboratories through a contract with the National Cancer Institute. BALB/c IFN-γ^−/−^ mice were obtained from Jackson Laboratories (Bar Harbor, ME). C57BL/6 mice with macrophages insensitive to IFN-γ (MIIG) were previously generated at Cincinnati Children’s Hospital Medical Center (30). *T*β*RIIf/f-Cre* mice were generated by crossing Floxed *T*β*RII* (*T*β*RIIf/f*) and *Ubc-CreERT2* (*Cre*) mice (14). To induce conditional deletion of *T*β*RII*, mice were injected intraperitoneally (i.p.) with 2 mg of tamoxifen (Sigma-Aldrich) in corn oil (Sigma-Aldrich) once daily for five consecutive days. Mice were treated with tamoxifen prior to intranasal (i.n.) allergen challenge. Animal care and experimental protocols were in accordance with the NIH *Guide for the Care and Use of Laboratory Animals* (81) and were approved by the Institutional Animal Care and Use Committee at Albany Medical College (protocol number 17-03006).

### A triple-challenge mouse model of AAD, primary influenza infection, and secondary S. pneumoniae infection.

For the OVA-AAD model, mice were immunized i.p. twice with 10 μg of OVA in 4 mg of aluminum hydroxide (General Chemical). The sensitized mice were anaesthetized with isoflurane and challenged i.n. with 100 μg of OVA in phosphate-buffered saline (PBS) once daily for 5 days. For induction of HDM-AAD, mice were anaesthetized and i.n. treated with 50 μg of HDM extract (Dermatophagoides pteronyssinus; Greer Laboratories) in PBS for three consecutive days every 3 weeks. Control non-AAD mice received 50 μl PBS. The AAD or non-AAD mice were infected i.n. with 10 PFU of H1N1 A/California/4/2009 (CA04) virus or H1N1 A/Puerto Rico/8/1934 (PR8) virus and subsequently infected with 2 × 10^2^ CFU of the S. pneumoniae serotype 3 A66.1 strain, 1.5 × 10^4^ CFU of the S. pneumoniae serotype 2 D39 strain, or 2 × 10^8^ CFU of methicillin-resistant Staphylococcus aureus (MRSA) strain USA300 at day 8 after influenza infection. Coinfection was routinely performed on week 1 after the last i.n. treatment, unless otherwise stated. This time point was chosen to minimize the unintended effects of i.n. PBS treatment while allowing investigation of the impact of AAD on host susceptibility to coinfection.

### Pulmonary viral and bacterial burdens.

Bronchoalveolar lavage fluid (BALF) was harvested by lavaging the lungs with 1 ml of PBS. Serial dilutions of cell-free BALF were added to MDCK cell monolayers and blood agar plates to enumerate viral PFU and bacterial CFU, respectively.

### Cytokine analysis.

Protein levels of IL-2, IL-4, IL-5, IL-6, IL-10, IL-12, IL-13, tumor necrosis factor alpha (TNF-α), and IFN-γ in cell-free BALF samples were analyzed using Bio-Plex mouse cytokine assays (Bio-Rad, Hercules, CA).

### Flow cytometric analysis.

The BALF cells were harvested in 1 ml of PBS. Live cells were enumerated based on trypan blue staining. Dead cells were labeled with fixable viability dye (FVD; eBioscience). Fc receptors were blocked by incubation with mouse 2.4G2 (FcγIII/II receptor) antibody. Fc receptor-blocked cells were then stained with mixtures of anti-mouse surface antigen mAbs: Alexa Fluor 488-conjugated anti-CD11b (clone M1/70; BioLegend), brilliant violet 421-conjugated anti-Ly6C (clone HK1.4; BioLegend), fluorescein isothiocyanate (FITC)-conjugated anti-CD4 (clone GK1.5; BD Pharmingen), phycoerythrin (PE)-conjugated anti-SiglecF (clone E50-2440; BD Pharmingen), PE-Cy7-conjugated anti-CD8 (clone 53-6.7; BD Pharmingen), FITC-conjugated anti-macrophage mannose receptor (clone C068C2; BioLegend), allophycocyanin-conjugated anti-CD11c (clone N418; BioLegend), peridinin chlorophyll protein (PerCP)-Cy5.5-conjugated anti-CD11b (clone M1/70; eBioscience), PerCP-Cy5.5-conjugated anti-F4/80 (clone BM8; BioLegend), and PE-Cy7-conjugated anti-Ly6G (clone 1A8; BioLegend). Stained cells were analyzed using a FACSCanto flow cytometer.

### Intracellular staining.

To enumerate GzmB^+^ T cells and IFN-γ^+^ T cells, 5 × 10^5^ live BALF cells were restimulated with CA04 virus at a multiplicity of infection (MOI) of 1 (5 × 10^5^ PFU/well) for 1 h, followed by 1 h of incubation with 10 μg/ml of brefeldin A (Sigma). Cells were then stained with FVD, FcR blocked, and cell surface stained as described above. This was followed by incubation with BD fixation/permeabilization solution. After washing with BD Perm/Wash buffer, the cells were intracellularly stained with a PE-conjugated anti-IFN-γ mAb (clone XMG1.2; BioLegend) or FITC-conjugated anti-granzyme B mAb (clone NGZB; eBioscience). Rat IgG1-PE and rat IgG2a-FITC were used as isotype controls. Stained cells were quantitated using a FACSCanto flow cytometer.

### *In vivo* phagocytosis assay.

FITC-labeled fluorescent beads were i.n. administered on day 7 after influenza infection. BALF phagocytic cells were analyzed for FITC fluorescence intensity 24 h later by flow cytometric analysis.

### *In vitro* bacterial burden assay.

BALF cells were harvested from non-AAD and AAD mice on day 8 after CA04 infection and cultured in 96-well plates with S. pneumoniae A66.1 at an MOI of 1. The culture supernatants were harvested and added to blood agar plates to enumerate bacterial CFU.

### Recombinant IFN-γ treatment.

Mice were given 20 μg of recombinant IFN-γ (BioLegend) i.n. on day 8 after influenza infection. One hour later, mice were i.n. inoculated with 10^5^ CFU of D39 with or without 20 μg of IFN-γ. The bacterial burden was then determined 4 h after D39 infection.

### Statistical analysis.

Results were analyzed using GraphPad Prism 6 software, with a *P* value of <0.05 considered to be statistically significant. Survival data were analyzed with a log rank (Mantel-Cox) test. All other data were analyzed by unpaired Student’s *t* test with Welch’s correction for comparison of two groups and by one- or two-way analysis of variance (ANOVA) with Bonferroni correction for comparison of multiple groups.
