# Supplementary figures and images for: Allergic Airway Disease Prevents Lethal Synergy of Influenza A Virus-Streptococcus pneumoniae Coinfection
Source: mBio. 2019 Jul 2;10(4):e01335-19. doi: 10.1128/mBio.01335-19 (PMC6606812; doi:10.1128/mBio.01335-19)

**A**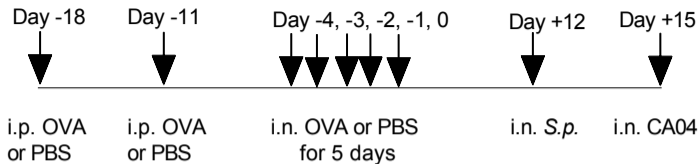

A66.1 → CA04

**B**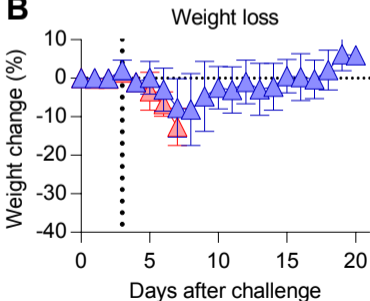**C**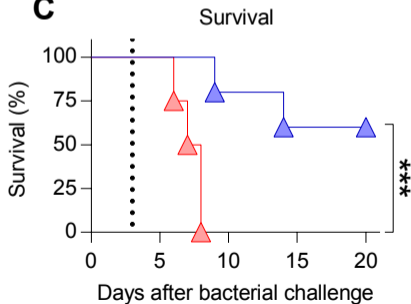

Non-AAD: A66.1 / CA04

OVA-AAD: A66.1 / CA04

Supplement: FIG S1 [file mBio.01335-19-sf001.pdf]

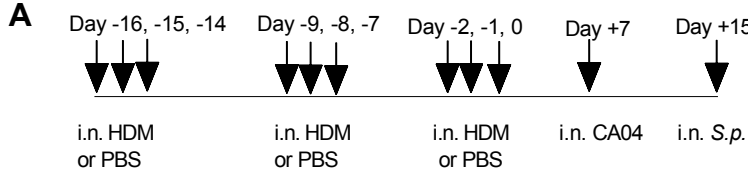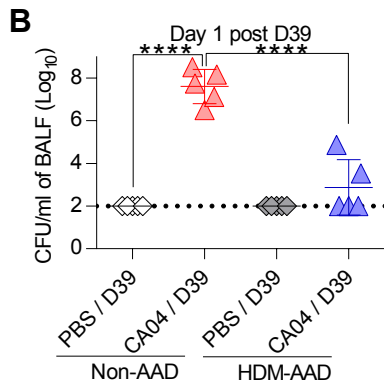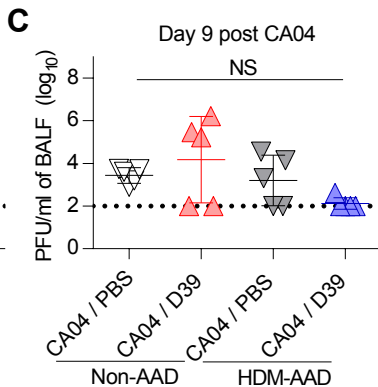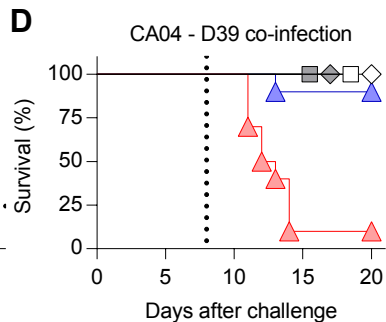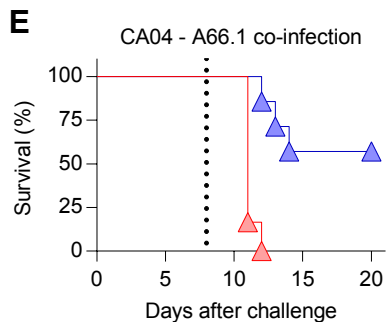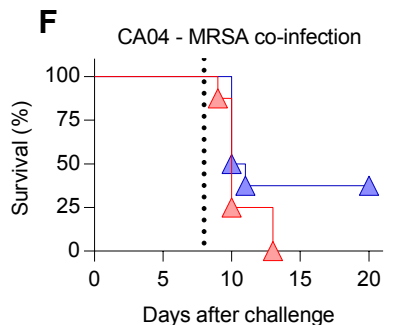

—△— Non-AAD: CA04 / A66.1

—▲— HDM-AAD: CA04 / A66.1

\*\*

—△— Non-AAD: CA04 / MRSA

—▲— HDM-AAD: CA04 / MRSA

\*\*\*\*

Supplement: FIG S2 [file mBio.01335-19-sf002.pdf]

# Co-infection in C57BL/6 strain

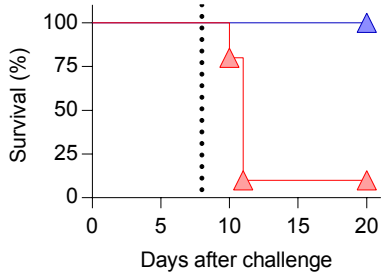

Non-AAD: CA04 / A66.1 \*\*  
OVA-AAD: CA04 / A66.1 \*\*

Supplement: FIG S3 [file mBio.01335-19-sf003.pdf]

## BALF for cytokine analysis

**A**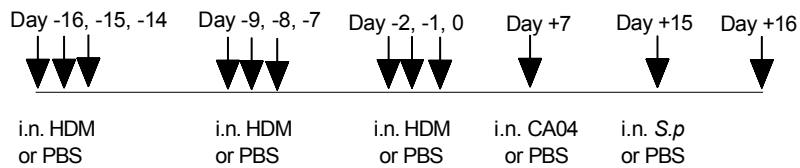**B**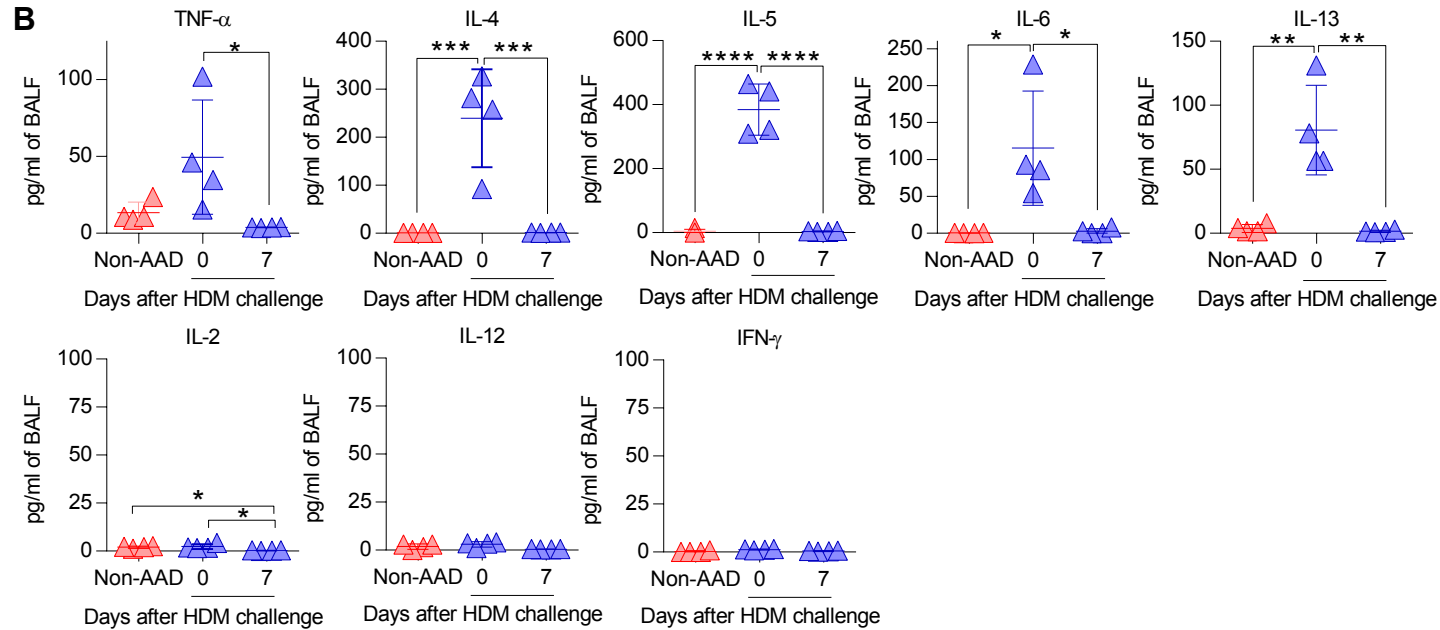**C**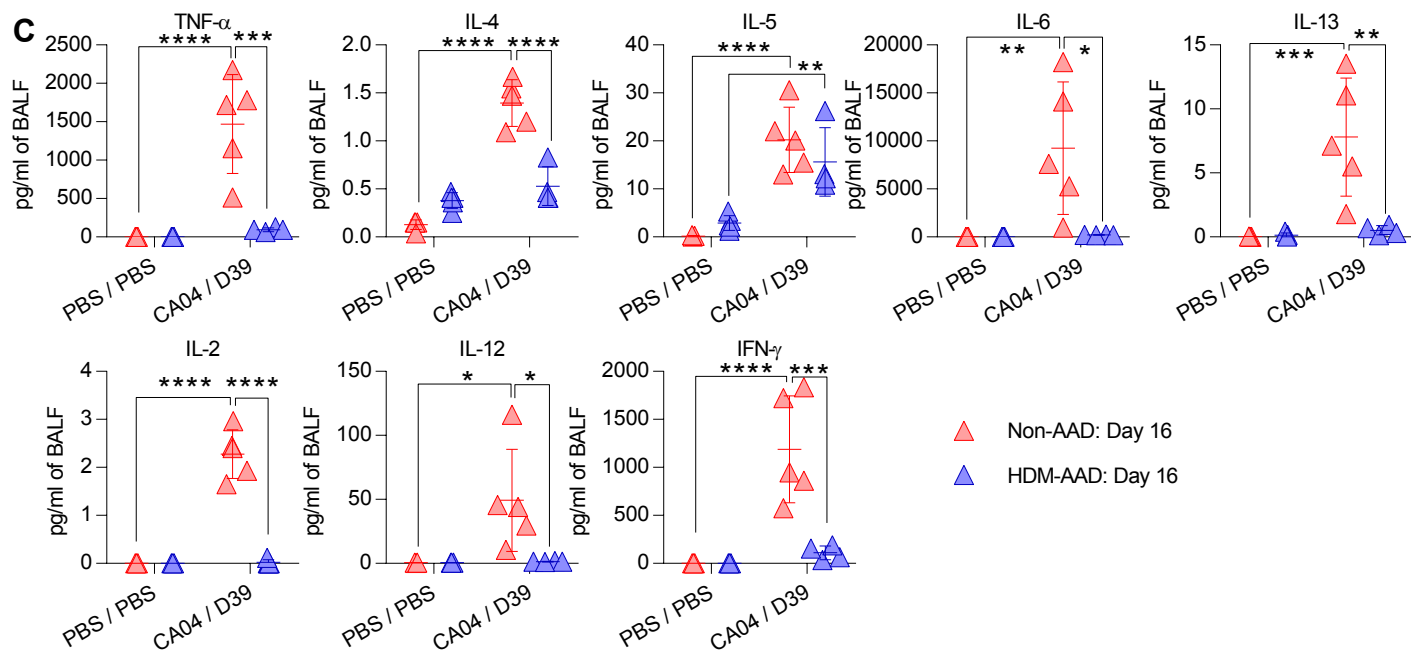

Supplement: FIG S4 [file mBio.01335-19-sf004.pdf]

**A**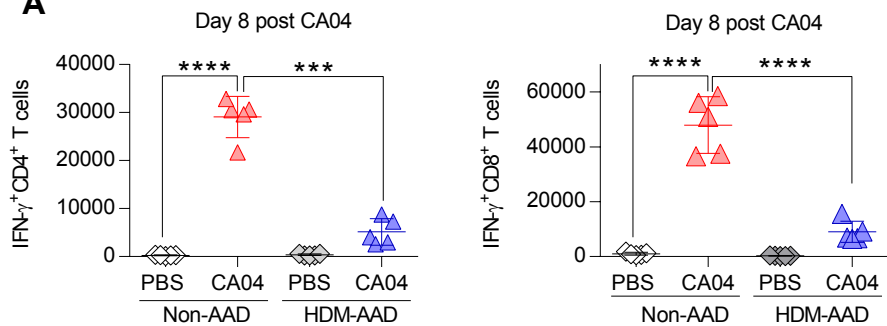**B**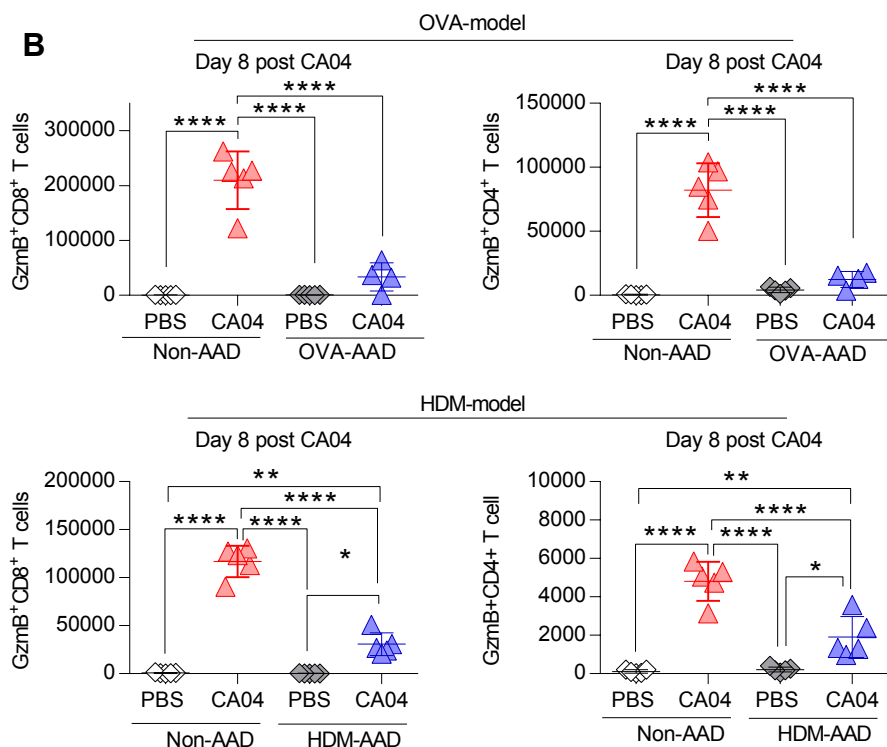**C**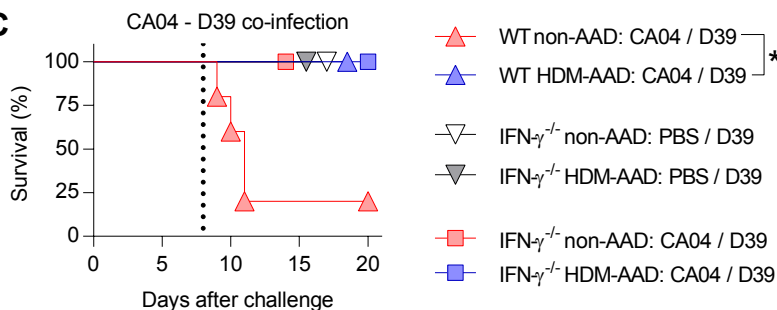

Supplement: FIG S5 [file mBio.01335-19-sf005.pdf]

**A**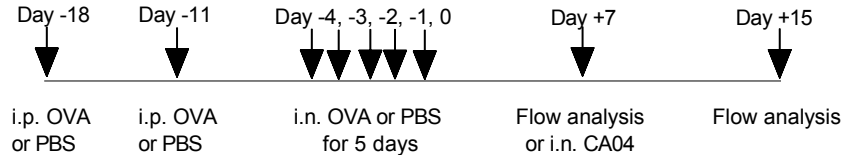**B**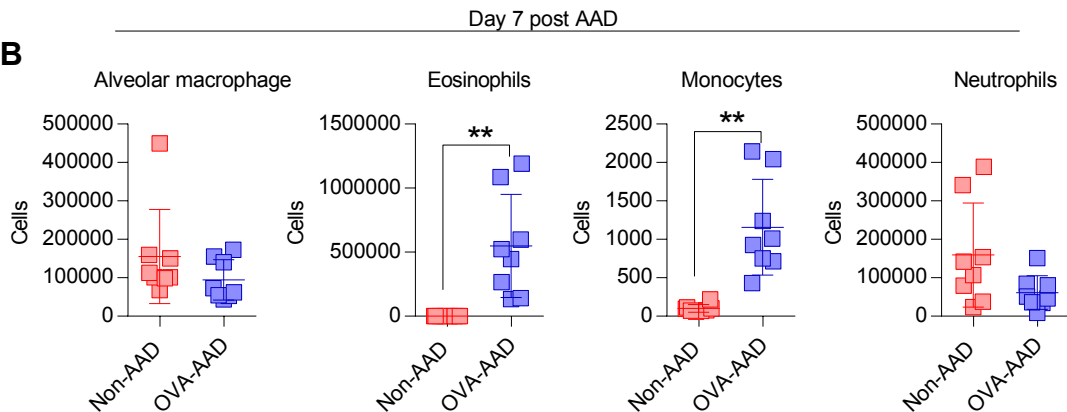**C**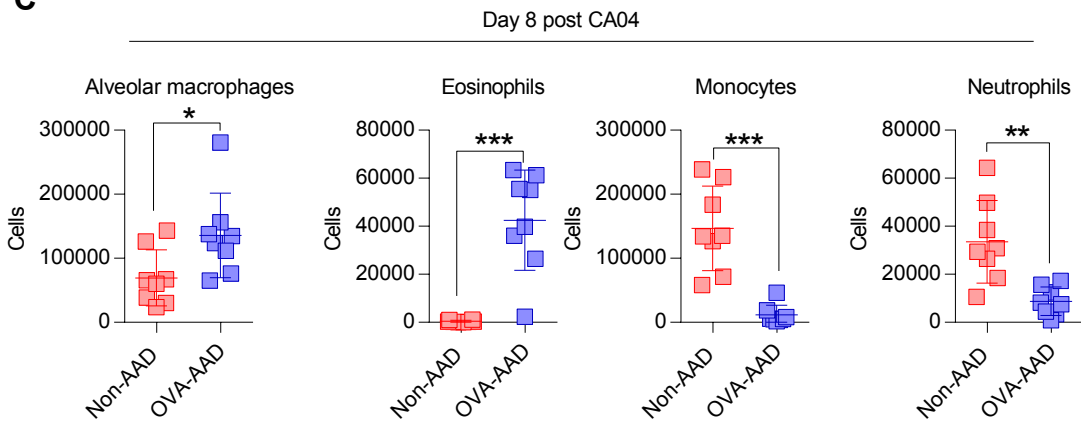

Supplement: FIG S7 [file mBio.01335-19-sf007.pdf]
