# Supplementary material for: Allergic Airway Disease Prevents Lethal Synergy of Influenza A Virus-Streptococcus pneumoniae Coinfection
Source: mBio. 2019 Jul 2;10(4):e01335-19. doi: 10.1128/mBio.01335-19 (PMC6606812; doi:10.1128/mBio.01335-19)
Supplement: FIG S6 [file mBio.01335-19-sf006.pdf]

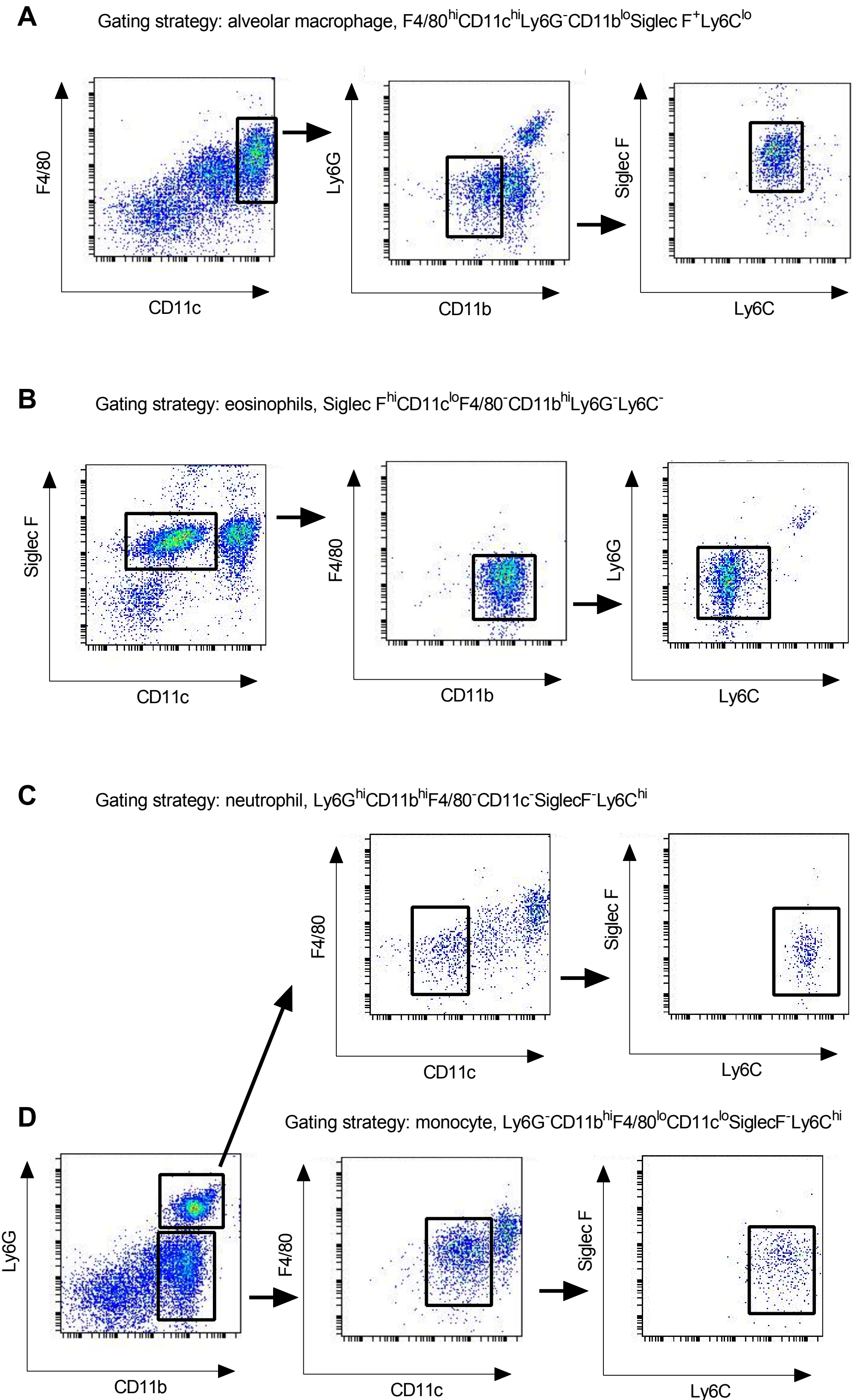
A
Gating strategy: alveolar macrophage, F4/80^{hi}CD11c^{hi}Ly6G^{-}CD11b^{lo}Siglec F^{+}Ly6C^{lo}
F4/80
CD11c
Ly6G
CD11b
Siglec F
Ly6C
B
Gating strategy: eosinophils, Siglec F^{hi}CD11c^{lo}F4/80^{-}CD11b^{hi}Ly6G^{-}Ly6C^{-}
Siglec F
CD11c
F4/80
CD11b
Ly6G
Ly6C
C
Gating strategy: neutrophil, Ly6G^{hi}CD11b^{hi}F4/80^{-}CD11c^{-}SiglecF^{-}Ly6C^{hi}
F4/80
CD11c
Siglec F
Ly6C
D
Gating strategy: monocyte, Ly6G^{-}CD11b^{hi}F4/80^{lo}CD11c^{lo}SiglecF^{-}Ly6C^{hi}
Ly6G
CD11b
F4/80
CD11c
Siglec F
Ly6C
