# Supplementary material for: Allergic Airway Disease Prevents Lethal Synergy of Influenza A Virus-Streptococcus pneumoniae Coinfection
Source: mBio. 2019 Jul 2;10(4):e01335-19. doi: 10.1128/mBio.01335-19 (PMC6606812; doi:10.1128/mBio.01335-19)
Supplement: FIG S8 [file mBio.01335-19-sf008.pdf]

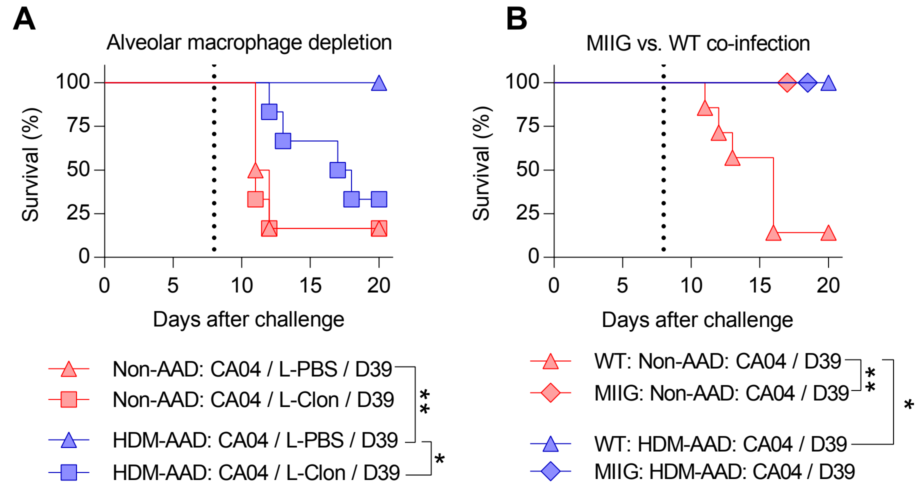

A
Alveolar macrophage depletion
Survival (%)
100
75
50
25
0
0
5
10
15
20
Days after challenge
Non-AAD: CA04 / L-PBS / D39
Non-AAD: CA04 / L-Clon / D39
HDM-AAD: CA04 / L-PBS / D39
HDM-AAD: CA04 / L-Clon / D39
**
*
B
MIIG vs. WT co-infection
Survival (%)
100
75
50
25
0
0
5
10
15
20
Days after challenge
WT: Non-AAD: CA04 / D39
MIIG: Non-AAD: CA04 / D39
WT: HDM-AAD: CA04 / D39
MIIG: HDM-AAD: CA04 / D39
**
*
