# Supplementary material for: Allergic Airway Disease Prevents Lethal Synergy of Influenza A Virus-Streptococcus pneumoniae Coinfection
Source: mBio. 2019 Jul 2;10(4):e01335-19. doi: 10.1128/mBio.01335-19 (PMC6606812; doi:10.1128/mBio.01335-19)
Supplement: FIG S9 [file mBio.01335-19-sf009.pdf]

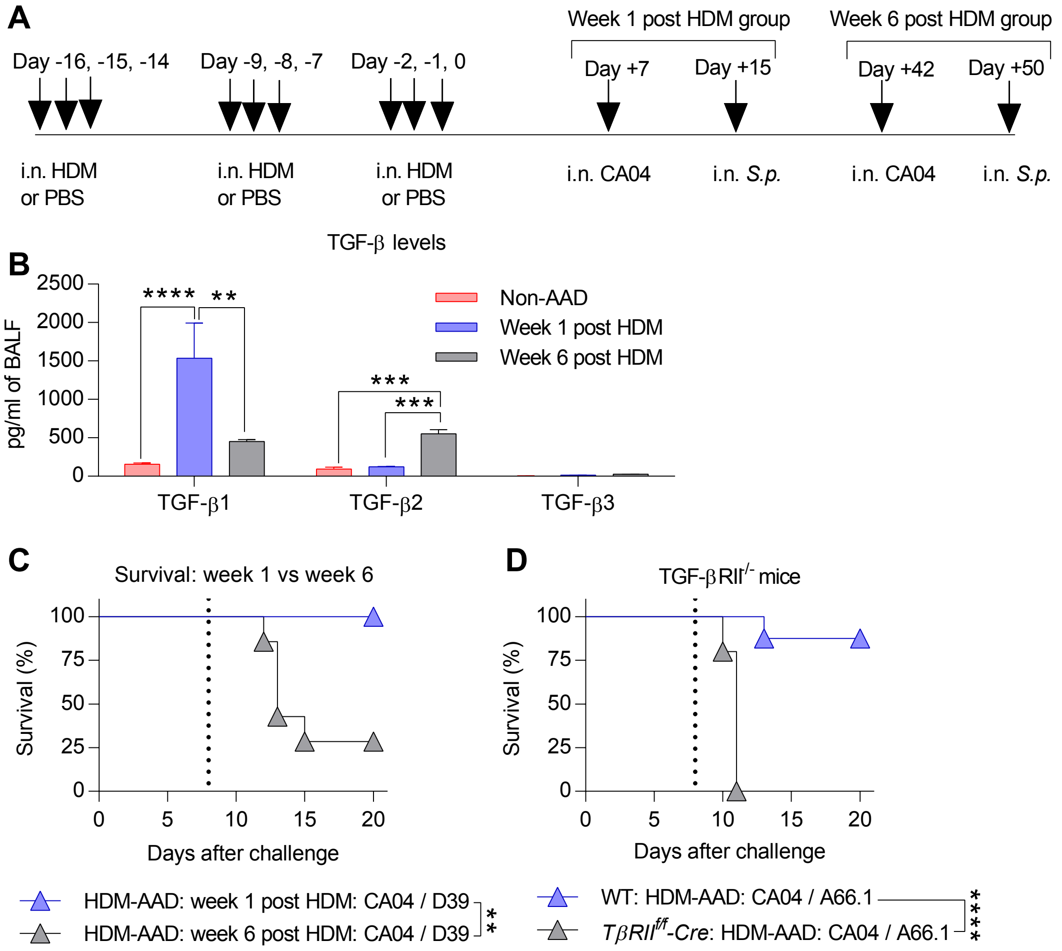

A
Week 1 post HDM group
Week 6 post HDM group
Day -16, -15, -14
Day -9, -8, -7
Day -2, -1, 0
Day +7
Day +15
Day +42
Day +50
i.n. HDM or PBS
i.n. HDM or PBS
i.n. HDM or PBS
i.n. CA04
i.n. S.p.
i.n. CA04
i.n. S.p.
B
TGF-β levels
pg/ml of BALF
2500
2000
1500
1000
500
0
****
**
***
***
Non-AAD
Week 1 post HDM
Week 6 post HDM
TGF-β1
TGF-β2
TGF-β3
C
Survival: week 1 vs week 6
Survival (%)
100
75
50
25
0
0
5
10
15
20
Days after challenge
HDM-AAD: week 1 post HDM: CA04 / D39
HDM-AAD: week 6 post HDM: CA04 / D39
**
D
TGF-βRII-/- mice
Survival (%)
100
75
50
25
0
0
5
10
15
20
Days after challenge
WT: HDM-AAD: CA04 / A66.1
TβRIIf/f-Cre: HDM-AAD: CA04 / A66.1
****
